# Animal Magnetism

**Published:** 1840

**Authors:** 


### ANIMAL MAGMETISM.

What has become of Rhode Island Animal Magnetism? We heard a report not long since, that it had broken out in Wheeling, Va. We hope it will float down the river to Cincinnati and Louisville. In a late number of the ***Boston Medical and Surgical Journal***, it is stated, that the French Academy had at their disposal, 3000 francs to present to the person, magnetized or not, who should read through an opake body, interposed between the eyes and the letters, with his eyes open or closed, but notwithstanding the hue and cry about ***clairvoyance***, not a single individual who pretended to read in that manner, has been able to do so before the committee of the Academy.

